# Cell-mediated and humoral immune profile to hydatidosis among naturally infected farm animals

**DOI:** 10.14202/vetworld.2020.214-221

**Published:** 2020-01-31

**Authors:** Faten A. M. Abo-Aziza, Seham H. M. Hendawy, Samah S. Oda, Dina Aboelsoued, Eman E. El Shanawany

**Affiliations:** 1 Department of Parasitology and Animal Diseases, Veterinary Research Division, National Research Centre, Cairo, Egypt; 2 Department of Pathology, Faculty of Veterinary Medicine, Alexandria University, Edfina, Egypt

**Keywords:** cell-mediated immune response, humoral immune response, hydatidosis

## Abstract

**Background and Aim:**

Cystic echinococcosis (CE) is a widespread parasitic disease caused by *Echinococcus granulosus* tapeworm infect different intermediate hosts including sheep, cattle, and camels. The intermediate host’s immune response to the hydatid cyst is still conflict and complex. The current study was designed to evaluate the immune response in sera of hydatid naturally infected sheep, cattle, and camels in the form of features of inflammatory cell infiltrations, levels of Th1 and Th2 cytokines, besides the humoral specific immunoglobulin G (IgG) responses.

**Materials and Methods:**

Thirty-nine sheep, 74 cattle, and 79 camels’ sera were collected and considered as CE naturally infected and ten samples from each species were graded as non-infected. Lung specimens were collected for histopathological examination. The quantitative concentrations of tumor necrosis factor-α, interleukin (IL)-6, IL-4, and IL-10 were determined. Different antigens were prepared from hydatid cyst; hydatid cyst fluid of lung origin hydatid cyst fluid of liver origin, hydatid cyst protoscoleces of lung origin (HCP-g), hydatid cyst protoscoleces of liver origin, hydatid cyst germinal layer of lung origin, and hydatid cyst germinal layer of liver origin; and characterized by gel electrophoresis and Western blotting analysis. The total specific IgG level against *E. granulosus* infection was measured using an indirect enzyme-linked immunosorbent assay.

**Results:**

The results indicated that the cellular immune response in the infected tissues was characterized by inflammatory cell penetration. The pro-inflammatory Th1 cytokine profile was predominant in infected animals in comparison with non-infected ones. However, the humoral immune response was seen as a high level of IgG in infected animals. The presented data approved that the HCP-g antigen could be considered as a delegate antigen for all other prepared antigens with an immunoreactive band at molecular weights 32 kDa.

**Conclusion:**

This study provides a fundamental insight into the events that manipulate cellular and humoral immune profiles in an intermediate host; sheep, cattle, and camel that naturally infected with CE. Hence, it was concluded that CE is a constant disease and confirm the reactivity Th1 in combating hydatid cyst. Besides, it could lead to the activation of the humoral immune response in the form of a high level of IgG.

## Introduction

Cystic echinococcosis (CE) or hydatidosis is a worldwide parasitic disease caused by the cestode *Echinococcus granulosus*. Hydatidosis is a silent cyclozoonotic disease of humans and herbivore animals causing high morbidity and mortality rate [1]. The larval stage of *E. granulosus* has a complex life cycle which alternates between definitive carnivore hosts such as dogs and other canids [2] and intermediate hosts including herbivore animals such as cattle, pigs, buffaloes, camels, sheep, and goats [3]. Furthermore, human may be infected as an intermediate host accidentally by contaminated food or water or by direct contact with infected dog feces [4]. Activated oncosphere larvae are released from hatched eggs at the gastrointestinal tract of the intermediate hosts. They penetrate the intestinal wall to reach the bloodstream and eventually, reside in the internal organs where they mature to form hydatid cysts [5]. The hydatid cysts develop in different viscera, especially in the liver and lungs, which are common places for cyst formation [6] and gradually grow from 1 cm to 5 cm a year [7]. The hydatid cysts are usually unilocular, fluid-filled bladder structures which consist of two layers; outer laminated layer surrounded by fibrous host tissue (pericyst) and innermost germinal layer where brood capsules and protoscoleces may bud from germinal membrane [8]. The host pericyst layers that surround the tissue of the parasite are considered as part of the cyst structures. These layers are of host origin and have an important role in the immunological response against the parasite [9]. The definitive hosts are infected after ingesting offal containing fertile hydatid cysts with viable protoscoleces which are released and reach the small intestine where they develop to adult worm after 4-5 weeks [9]. Recent clinical cyst classifications have underlined that hydatid cysts are morphologically different [8].

The establishment of hydatid cysts within the intermediate host occurs in long-term growth, so different immune mechanisms are induced during host-parasite interplay [10]. The early immune response toward CE was found to be not successful in preventing the infection and this implies the existence of elaborated by *E. granulosus* [11]. *E. granulosus* displays different immunological relationships with its hosts, therefore, great efforts have been invested to understand the immunobiology of the parasite in the intermediate host [7]. T-helper is the main immunocompetent cells by secretion of immune mediators Th1/Th2 [12]. The early stage of CE is characterized by immunoglobulin G (IgG) response that plays a crucial role in the killing of larval metacestode [10]. In addition, the host immune responses usually depend on infiltrated cells and low-level of polarized Th1 responses with low production of pro-inflammatory cytokines. However, Th2 polarized immune response produces anti-inflammatory cytokine with the progression of the cyst [13]. Moreover, interleukin (IL)-10 orchestrates the chronic stage of CE which is modulated by protoscoleces developmental stages [8]. Furthermore, the cellular inflammatory infiltrations including neutrophils, lymphocytes, and macrophages are considered as a characteristic feature of echinococcal infection [10]. Moreover, CE infection is remarkable with mixed Th1/Th2 polarized cytokines [8]. Rostami-Rad *et al*. [14] found that the Th1-type cytokine profile was predominant at the early post-infection phase (3-4 weeks), however, the shift to Th2-type cytokine appeared in the 4^th^ week and this suggests that the shift in Th1-type cytokine profile toward Th2-type explains that CE is a constant disease and confirm the reactivity Th1 in combating *E. granulosus* infection.

In Egypt, CE exists in various intermediate hosts, including cattle, buffaloes, sheep, and camels [15]. Unfortunately, there are limited reports about the host-parasite interplay and immunopathogenesis for the course of the infection in naturally infected animals. Understanding the immune response against this disease is urgently needed to find new biomarkers for diagnostic and prognostic purposes that will be a benefit in the treatment and control of such *E. granulosus* infections in farm animals.

The current study was designed to evaluate the immune response in sera of hydatid naturally infected sheep, cattle, and camels in the form of features of inflammatory cell infiltrations, evaluate levels of Th1 and Th2 cytokines, besides the humoral specific IgG responses. Furthermore, the characterization of hydatid cyst different prepared antigens; protoscoleces, hydatid cyst fluid, and germinal layer of camel origin was done as an example.

## Materials and Methods

### Ethical approval

This study was approved by the Medical Research Ethics Committee of National Research Centre, Egypt (Number: 16230). The experiments were conducted according to the guidelines established by the International Animal Ethics Committee and according to the local laws and regulations.

### Animals and samples collection

Tissue samples with their corresponding blood were collected from 397 sheep, 401 cattle, and 341 camels presented for slaughter at El-Warak, El-Basatin, and El-Moneibs laughterhouses from April 2017 to June 2018. All samples of each species were classified into two groups infected and non-infected according to postmortem examination. Thirty-nine sheep, 74 cattle, and 79 camels were considered as naturally infected with CE and ten animals from each species were graded as non-infected based on the absence of visible CE infection and clinical signs. Sera were aliquoted and stored at −20°C until use. Liver and lung specimens were collected and rapidly fixed for histopathological examination. Hydatid cysts of camel origin were collected in phosphate-buffered saline (PBS) PH, 7.4 for the preparation of antigens.

### Histopathological examination

Lung tissue specimens from infected animals were fixed rapidly in 10% neutral buffered formalin for about 24 h. Fixed specimens were trimmed, washed, dehydrated in ascending grades of ethyl alcohol, cleared in xylene, and embedded in paraffin wax. Then, thin sections of 4-5 µm thickness were performed and stained with hematoxylin and eosin for general microscopic examination [16].

### Evaluation of Th1-polarized cytokine (tumor necrosis factor [TNF]-α, IL-6), and Th2-polarized cytokine (IL-4, and IL-10)

Sandwich enzyme-linked immunosorbent assay (ELISA) was utilized to measure serum cytokine levels. TNF-α, IL-6, IL-4, and IL-10 concentrations were determined with commercially available kits (Sigma-Aldrich, USA) following the manufacturer’s instructions. The color change was measured by a spectrophotometer at a wavelength of 450 nm.

### Preparation of hydatid cyst antigens

Collected hydatid cysts from infected lungs and livers of the slaughtered camels were used for preparing different hydatid cyst antigens; hydatid cyst fluid of lung origin (HCF-g), hydatid cyst fluid of liver origin (HCF-v), hydatid cyst protoscoleces of lung origin (HCP-g), hydatid cyst protoscoleces of liver origin (HCP-v), hydatid cyst germinal layer of lung origin (HCG-g), and hydatid cyst germinal layer of liver origin (HCG-v) [17]. Hydatid cyst fluid was aseptically aspirated and centrifuged at 5000× *g* for 20 min in a cooling centrifuge. The supernatants were collected, dialyzed, and stored as HCF-g and HCF-v antigens at −20°C. Protoscoleces were obtained from the sediment of collected cyst fluid. They were washed 3 times with PBS, pH 7.2, and resuspended in PBS. The protoscoleces were exposed to three cycles of freezing, thawing, and sonication (1 min and 0.5 amplitude), then they were microscopically checked until no intact protoscoleces were detected. Then, the antigens were centrifuged at 10,000× *g* for 30 min and supernatants were collected and stored as HCP-g and HCP-v antigens at −20°C. Germinal layers of hydatid cysts were separated, washed several times and homogenized in PBS, pH 7.2. The homogenate was sonicated for three cycles (1 min and 0.5 amplitude) and centrifuged at 10,000× *g* for 30 min in a cooling centrifuge. The supernatants were aspirated and stored as HCG-g and HCG-v antigens at −20°C. The total protein content of different hydatid cyst antigens was measured according to Lowry *et al*. [18].

### Gel electrophoresis and Western blotting analysis

All the hydatid cyst prepared antigens were individually fractionated in four 12% sodium dodecyl sulfate-polyacrylamide gel electrophoresis (SDS-PAGE) using pre-stained molecular weights (MW) protein marker 16-270 kDa (GeneDirex, USA). After SDS-PAGE, one gel was stained with Coomassie Brilliant Blue R-250 dye and analyzed using a gel documentation system [19]. The others were blotted on 0.45 nitrocellulose membranes [20]. Briefly, the blocked membranes with 1% dry skimmed milk/Tris-buffered saline (TBS) were incubated overnight with positive naturally infected sera from camel, cattle, and sheep origins at dilution 1:50 in TBS/0.5% bovine serum albumin (BSA) against all prepared antigens. The nitrocellulose sheets were probed for 1 h with protein-A peroxidase conjugate at 1:2500 diluted with 0.5% BSA/TBS buffer. The substrate solution (1-chloronaphthol, Sigma-Aldrich, USA) was used for 20 min to develop the immunogenic bands. Then, the nitrocellulose membranes were photographed and analyzed using Molecular Imager Gel Doc™ XR+ with Image Lab Software (Bio-Rad, California, USA).

### Diagnostic accuracy of the selected antigen

The prevalence of hydatid cyst infection, sensitivity, and specificity of HCP-g antigen was calculated in contrast to the parasitological investigation of the examined camel sera [12].

### *E. granulosus* specific IgG assay

Evaluation of IgG antibody levels against *E. granulosus* infection was performed using indirect ELISA (I-ELISA). Checkerboard titrations were used for the determination of the optimal concentration of antigens, tested sera dilutions, and conjugate dilution. The assay was performed according to the method previously described [21]. Briefly, the plates were coated with diluted antigen at the concentration of 4 µg/ml in coating buffer; carbonate-bicarbonate buffer, pH 9.6; (100 µl/well), and incubated at 4°C overnight. The unbinding sites were blocked using 1% BSA in coating buffer then incubated for 1 h at room temperature. After washing, 100 µl diluted sera (1:50) of samples in 0.05% PBS-T-20 were added and incubated for 2 h at 37°C. The plates were washed and protein-A horseradish peroxidase conjugate was added (100 µl/well) at dilution 1:1000 and incubated for 1 h at 37°C. Then, the substrate orthophenylenediamine was added and the optical densities (OD) were read at a wavelength of 450 nm with an ELISA reader (BIO-TEK, INC, ELx, 800UV, USA). The cutoff point of OD values was determined as described by Almazán *et al*. [22]. Sera obtained from healthy; non-infected camels were used in the assay as a negative control.

### Statistical analysis

The data were analyzed using a one-way analysis of variance. Results were expressed as mean ± standard deviation and values of p<0.05 and p<0.01 were considered statistically significant and highly significant respectively. The curves were represented by MEDCALC easy-to-use statistical software.

## Results

**Figure-1:**
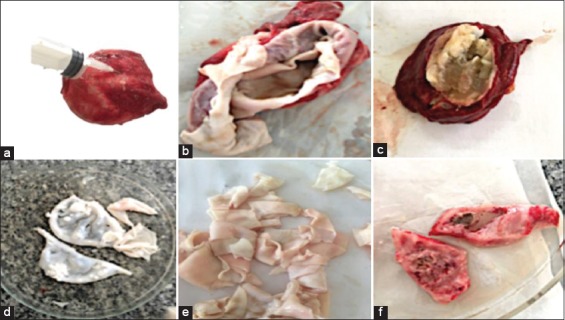
Hydatid cyst of camel showing aspiration of the fluid from the cyst (a), opened evacuated hydatid cyst germinal layer of cyst wall that consisted of an outer thick fibrous layer and an inner thin germinal layer (b), opened hydatid cyst of cattle with semi-solid matrix (c), a germinal layer of hydatid cyst of camel liver (d and e), tissue from hydatid infected lung of sheep (f).

As shown in Figure-1, hydatid cyst of camel showing aspiration of the fluid from the cyst (Figure-1a), opened evacuated hydatid cyst germinal layer of cyst wall. The cyst wall consisted of an outer thick fibrous layer and an inner thin germinal layer (Figure-1b) and opened hydatid cyst with a semi-solid matrix (Figure-1c). The hydatid cyst was full of scolices and membranes which replace the hydatid liquid. Germinal layer (Figure-1d and e) and tissue from hydatid infected lung of cattle (Figure-1f).

### Histopathological findings

**Figure-2:**
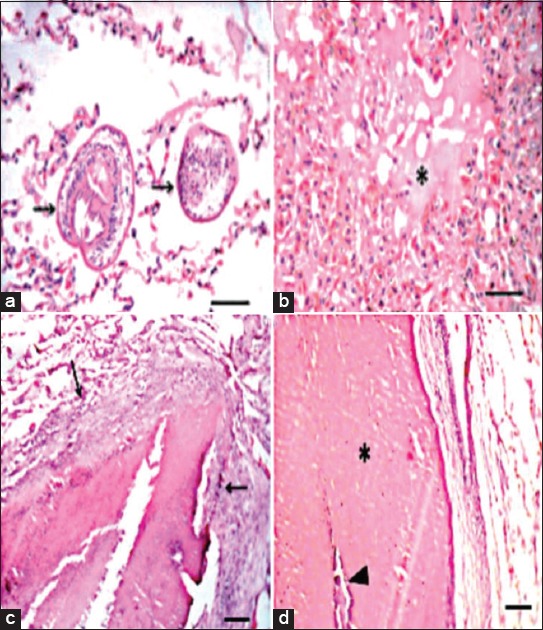
Lung of hydatid cyst infected camel showing: (a) Presence of scoleces (arrows) in alveoli, Bar = 50 µm, (b) pulmonary edema (asterisk), Bar = 50 µm, (c) suppurated cyst (abscess), Bar = 100 µm, (d) caseated (asterisk) and calcified (arrowhead) cyst, Bar = 100 µm.

Lungs of camels had marked hydatid cysts that were consisted of the outer laminated layer and fibrous layer penetrated with inflammatory cells. The surrounding pulmonary alveoli were collapsed and some were emphysematous. Scoleces were found in alveoli (Figure-2a) with mononuclear inflammatory cells and few multinucleated giant cells aggregation. Furthermore, there were severe congestion, hemorrhage, and edema (Figure-2b). One cyst was suppurated (Figure-2c) and others were caseated (Figure-2c).

### Th1/Th2-type cytokine profile in hydatid naturally infected-animals

The serum level of Th-1 pro-inflammatory cytokines, TNF-α and IL-6 and Th-2 anti-inflammatory cytokines, IL-4 and IL-10, in all the animals, was determined by sandwich ELISA, and the results obtained are presented in Table-1. The data revealed that infected sheep showed significantly a higher serum level of TNF-α and IL-6 in comparison with the corresponding non-infected ones. However, IL-4 serum level was significantly (p<0.05) lower than that of non-infected sheep. Hydatid infected cattle demonstrated a significantly (p<0.05) higher serum level of Th-1 pro-inflammatory cytokine, TNF-α and IL-6, comparing to non-infected cattle. However, Th-2 anti-inflammatory cytokines (IL-4 and IL-10) serum level was significantly lower (p<0.05) than that of non-infected cattle. Data revealed that Th1-polarized cytokines (TNF-α and IL-6) serum levels were significantly higher (p<0.05) in hydatid infected camel comparing to non-infected (Table-1).

**Table-1:** Serum levels of Th1-polarized cytokines (TNF-α and IL-6) and Th2-polarized cytokines (IL-4 and IL-10) in hydatid infected-sheep cattle and camels (ng/ml).

Parameter/Animals	Sheep		Cattle		Camel	
		
	Non-infected	Hydatid cyst infected	Non-infected	Hydatid cyst infected	Non-infected	Hydatid cyst infected
TNF-α	8.41±1.24	12.04±0.712*	9.29±0.647	13.844±0.316*	10.76±0.289	16.84±0.316**
IL-6	9.10±0.51	13.62±0.954*	7.83±0.587	10.361±1.370*	8.38±0.741	13.39±0.459**
IL-4	9.76±0.873	6.39±0.647*	13.29±0.734	8. 472±0.312*	12.78±0.548	10.84±0.975
IL-10	11.32±0.755	9.57±0.289	16.28±1.281	10.589±0.921*	11.48±0.673	9.81±0.762

All data expressed as Mean±SE.

* and

** Significantly different than non-infected group at p<0.05 and p<0.01, respectively. TNF-α=Tumor necrosis factor-alpha, IL=Interleukin

### Electrophoretic and immunoreactive bands profile of hydatid cyst

**Figure-3:**
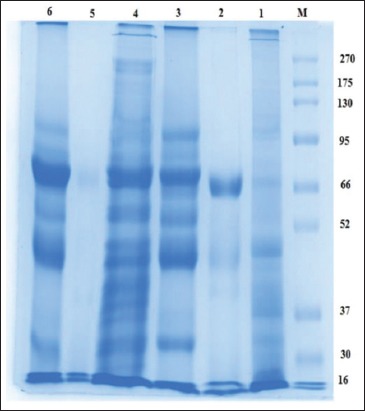
Sodium dodecyl sulfate-polyacrylamide gel electrophoresis electrophoretic protein profile of hydatid cyst protoscoleces-lung camel origin (HCP-g, Lane 1), hydatid cyst fluid-lung camel origin (HCF-g, Lane 2), hydatid cyst germinal layer-lung camel origin (HCG-g, Lane 3), hydatid cyst protoscoleces -liver camel origin (HCP-v, Lane 4), hydatid cyst fluid-liver camel origin (HCF-v, Lane 5), hydatid cyst germinal layer-liver camel origin (HCG-v, Lane 6), and BLUltra prestained protein ladder, GeneDirex (Lane M, 16-270 kDa).

Overall polypeptide bands exhibited by the different prepared antigens; HCP-g, HCF-g, HCG-g, HCP-v, HCF-v, and HCG-v were 7, 3, 5, 16, 2, and 5 bands in number at MW 270, 103, 68.7, 47.7, 37.5, 32.8, and16 kDa; 65.8, 46.0, and 16.2 kDa; 98.9, 70.7, 54.0, 45.6, and 32.1 kDa; 270, 250.9, 223,175, 151.3, 112.1, 97.6, 70.9, 55.7, 48.1, 45.5, 41, 38.5, 36.4, 32, and 23 kDa; 70.9, and 20 kDa; and 105.1, 73.2, 55.8, 46.5, and 31.8 kDa, respectively (Figure-3). The resultant data revealed four shared protein bands at MW 270, 32, 45, and 46 between HCP-g and HCP-v; HCP-g, HCG-g, HCP-v, and HCG-v; HCG-g and HCP-v; and HCF-g and HCG-v, respectively (Figure-3).

**Figure-4:**
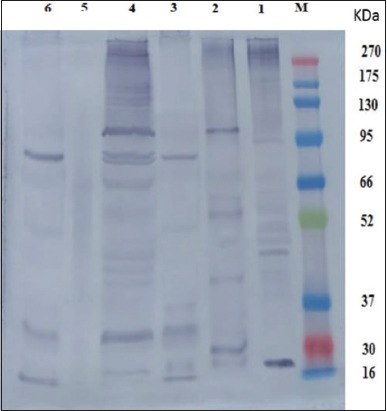
Western blot analysis profile of hydatid cyst protoscoleces-lung camel origin (HCP-g, Lane 1), hydatid cyst fluid-lung camel origin (HCF-g, Lane 2), hydatid cyst germinal layer-lung camel origin (HCG-g, Lane 3), hydatid cyst protoscoleces-liver camel origin (HCP-v, Lane 4), hydatid cyst fluid-liver camel origin (HCF-v, Lane 5), hydatid cyst germinal layer-liver camel origin (HCG-v, Lane 6) against pooled positive naturally infected camel sera and BLUltra prestained protein ladder, GeneDirex (Lane M, 16-270 kDa).

The reactive band profiles presented by binding of specific IgG in positive naturally infected sera against hydatid cyst antigens; HCP-g, HCF-g, HCG-g, HCP-v, and HCG-v were identified at MW 270, 190, 94.7, 70.3, 62.5, 56, 50.6, 48, 45, 42, 38.1, and 20.1 kDa; 103.2, 71.5, 59.1, 54.7, 41, 29.1, and 21.8 kDa; 99, 82.6, 50.8, 36.7, 32, 31.2, 19.5, and 16 kDa; 270, 155.2, 129.6, 122.6, 102.5, 83.7, 79.7, 65.8, 55.8, 45, 42, 40.7, 37.5, 32, and 17.9 kDa; and 100.4, 83.5, 50.5, 32, and 16 kDa, respectively. However, HCF-v did not show any reaction with naturally infected sera (Figure-4). The shared immune reactive polypeptides were detected at MW 50, 45, 42, and 32 kDa among HCP-g, HCG-g, and HCG-v; HCP-g and HCP-v; HCP-g and HCP-v; and HCG-v, HCP-v, and HCG-g, respectively (Figure-4).

**Figure-5:**
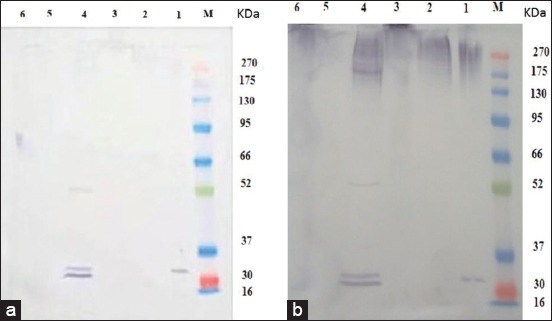
Western blot analysis profile of immunogenic bands of hydatid cyst antigens from camel origin against positive naturally infected cattle (a) and sheep sera (b). Hydatid cyst protoscoleces-lung camel origin (HCP-g, Lane 1a and b), hydatid cyst fluid-lung camel origin (HCF-g, Lane 2a and b), hydatid cyst germinal layer-lung camel origin (HCG-g, Lane 3a and b), hydatid cyst protoscoleces-liver camel origin (HCP-v, Lane 4a and b), hydatid cyst fluid-liver camel origin (HCF-v, Lane 5a and b), hydatid cyst germinal layer-liver camel origin (HCG-v, Lane 6a and b) against pooled positive naturally infected cattle (a) and sheep (b) sera and BLUltra prestained protein ladder, GeneDirex (Lane M, 16-270 kDa).

The reactive profile of immunogenic bands presented when such hydatid cyst antigens of camel origin verified against pooled positive naturally infected cattle and sheep sera revealed the immunoreactive band at MW 32 kDa with HCP-g antigen, while HCP-v antigen showed the bands at MW 50, 31, and 30 kDa and 53, 33, and 32 kDa when using positive naturally infected cattle and sheep sera, respectively (Figure-5a and b).

### Diagnostic accuracy of HCP-g antigen

HCP-g antigen was evaluated in the detection of hydatid cyst infection in collected camel sera using indirect ELISA. The used antigen achieved 75% sensitivity and a limited specificity 33%. The apparent prevalence of hydatidosis in tested serum samples was 69%.

### Detection of the specific IgG levels

**Figure-6:**
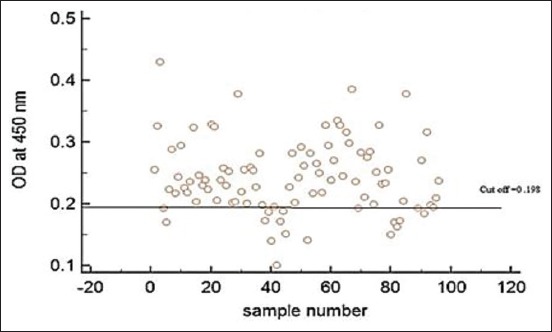
Total immunoglobulin G (IgG) antibody responses against a crude antigen preparation from *Echinococcus* *granulosus* protoscoleces was detected by an indirect enzyme-linked immunosorbent assay. The cutoff is the mean ± 3 SD of negative sera from non-infected camels. The figure illustrates elevation in the level of IgG within naturally infected animals in comparison with non-infected ones.

The total IgG level was determined to detect the humoral antibody responses to echinococcal infection. The IgG level was high in all infected animals in comparison with the non-infected ones (Figure-6).

## Discussion

*E. granulosus* could be considered as a basic cause of high rates of liver and lung condemnation in slaughtered farm animals [23]. Urgent insights into host-metacestode interaction, immune evasion and immunopathogenesis of larval metacestode (hydatid cyst formation) are needed for diagnostic improvement and monitoring progression/regression of this disease.

In the current study, the lungs of camels had marked hydatid cysts which consisted of the outer laminated layer and fibrous layer penetrated with inflammatory cells. The surrounding pulmonary alveoli were collapsed, and some were emphysematous. Scoleces were found in alveoli with mononuclear inflammatory cells and few multinucleated giant cells aggregation. The cellular reaction associated with hydatid cysts was indicative of delayed hypersensitivity reactions [24]. The observed lesions associated with hydatid cysts in the examined lung tissues were similar to those reported before [23].

The echinococcal infection can stimulate or suppress immune responses, which persist and flourish for a long time in their mammalian hosts [25]. Th1 and Th2 cytokines induced different immune pathways to fight parasitic infections; Th1 cytokines coordinate cellular immune responses, and Th2 cytokines coordinate humoral immune responses [12]. In the current study, the serum levels of Th-1 pro-inflammatory cytokines, TNF-α and IL-6, in infected sheep, cattle, and camel were significantly higher in comparison with their levels in non-infected ones. However, Th-2 anti-inflammatory cytokines, IL-4 and IL-10, serum levels in infected sheep, cattle, and camel were lower than that of non-infected ones. These results showed a pattern of increase in Th1-type cytokines and decrease in a Th2-type cytokine. This agreed with results of Rostami-Rad *et al*. [14] which revealed a pattern of increase in Th1-type cytokines and decrease in a Th2-type cytokine after protoscoleces inoculation in experimentally infected mice at the beginning of the infection, however, after several weeks post-infection, Th1-type cytokines decreased and a Th2-type cytokine increased. Furthermore, these results are consistent with former studies that implied the concurrent activation of type 1 and type 2 cytokines in response to hydatid antigens [10]; however, the predominance of Th2 profiles in peripheral immune reactions may be reported in found hydatid infection in human hosts [26]. This could be attributed to dominant Th1 cytokines usually found to be associated with the killing and clearance of protoscoleces [7]. As well, regulatory mechanisms which are elevated gradually with the prolonged chronicity are responsible for inhibition of metacestode killing and immunoregulation of effector cells to limit the inflammatory responses [13] as it is able to control both Th1 and Th2 parasite killing effector mechanisms [11].

Modulation of the host immune response may also be relevant for selection toward commensalism, since it may prevent deleterious effects to the host resulting from the exacerbated immune response [27]. In general, the presented results showed a mixed coexistence of Th1 and Th2 with largely Th1 dominance. The immunological events related to this type are characterized by Th-1 polarized cytokine which is involved in initiating calcification [28] with a high level of macrophages at host pericyst layer [29]. The presented result confirmed this observation as showed a calcified cyst with macrophages infiltration in the surrounding tissue of the hydatid cyst in infected animals. However, the presence of eosinophils was able to kill protoscoleceswith Th2 polarized cytokine [30]. The coexistence of Th1 and Th2 response is a remarkable feature of echinococcal infection. This is maybe due to the presence of different CE antigens with distinct epitopes to both types of T cells. Cell-mediated immunity is a characteristic feature of echinococcal infection [26,31].

In the present study, a comprehensive investigation to immunoreactive protein expression patterns of different prepared hydatid cyst antigens of camel origin; HCP-g, HCF-g, HCG-g, HCP-v, HCF-v, and HCG-v are electro-blotted against their homologous serum (positive naturally infected-hydatid cyst camel) and heterologous sera of positive naturally infected-hydatid cyst cattle and sheep sera. The resultant data revealed that HCP-g antigen could be considered a representative antigen for all other prepared antigens, where, such antigen shared them in one or more immune reactive polypeptide bands detected at MW 50, 45, 42, and 32 kDa among HCP-g, HCG-g, and HCG-v; HCP-g and HCP-v; HCP-g and HCP-v; and HCG-v, HCP-v, and HCG-g, respectively, using their homologous serum. Moreover, Burgu *et al*. [32] blotted HCF from sheep origin and tested it against sheep infected sera found immune responses to antigenic bands at 116 kDa. This difference may be attributed to the difference in hydatid cyst origin, different quantity and quality of used chemical reagents [33].

The results revealed a limited specificity (33%), which could be attributed to the failure of HCP-g antigen in the detection of true negative animal cases in non-infected hydatid cyst camels’ sera. In addition, HCP-g antigen recorded a good sensitivity (75%) and apparent prevalence (69%) which may be attributed to the common shared immunoreactive bands which exhibited at MW 32 kDa by HCP-g antigen and may be responsible for detecting high number of true positive cases in parasitological infected hydatid cyst camels’ sera. Similar results were obtained by El-Shanawany *et al*. [34] who found that the immunoreactive bands of HCG from camel origin were 30, 49, 77, and 99 kDa and this was similar to the current study in finding 32 kDa reacted with naturally camel serum sample. As well as, the immunodominant protein band at MW 32 kDa was detected with HCP-g antigen using the heterologous sera against all different prepared hydatid cyst antigens of camel origin. This representative trait of HCP-g antigen could be attributed to the life stage universality dynamic nature of protoscoleces proteomic analysis [35] where our present results revealed the presence of the same shared immunoreactive band at 32 kDa with HCP-v antigen when the heterologous serum of sheep origin was used. Consequently, these data explore the potentiality of HCP-g antigen to be verified as a candidate vaccinal antigen to overcome hydatidosis in farm animals with great concern to the band at MW 32 kDa which could also be used as a protein probe for sensitive detection of CE in herbivorous animals.

The current results indicated an elevation in the total IgG levels in infected camels comparing with non-infected ones. Furthermore, Rostami-Rad *et al*. [14], the total IgG level started to increase after 2 weeks’ post-inoculation in experimentally infected mice, which is a representative of the humoral response against the parasite. In general, echinococcal infection could lead to activation of the humoral immune response in the form of a high level of IgG, IgM, and IgE [10].

## Conclusion

CE naturally infected farm animals developed cellular and humoral immune responses. The results showed a pattern of increase in Th-1 type cytokines and decrease in Th-2 type cytokines. This could explain that CE is a constant disease and confirm the reactivity Th1 in combating *E. granulosus* infection. In addition, echinococcal infection could lead to activation of the humoral immune response in the form of a high level of IgG.

## Authors’ Contributions

FAMA designed the study, performed the immunological analysis, and contributed to laboratory work analysis, statistics, data interpretation, manuscript preparation, and corresponded the authorship. SHMH contributed to antigens preparation, gel electrophoresis and Western blotting analysis with their interpretation. SSO performed the histopathological examination. DA shared in the preparation of antigens. EEE shared in gel electrophoresis and Western blotting analysis. All authors assisted in manuscript preparation and have read and approved the final manuscript.
